# Commentary on “Mechanistic insights into PFOS-induced inflammatory bowel disease: a network toxicology and molecular docking study”

**DOI:** 10.1097/JS9.0000000000002947

**Published:** 2025-07-09

**Authors:** Xiaolin Hu, Ze Xiang, Hua Zhu

**Affiliations:** a Department of Gastroenterology, Affiliated Hospital 6 of Nantong University, Yancheng Third People’s Hospital, Yancheng, Jiangsu, China; b Affiliated Yancheng Hospital, School of Medicine, Southeast University, Yancheng, Jiangsu, China; c Zhejiang University School of Medicine, Hangzhou, China

*Dear Editor,*

We read with interest the recent study by Wang *et al*, entitled “Mechanistic insights into PFOS-induced inflammatory bowel disease: a network toxicology and molecular docking study”[1]. This investigation, which integrated network toxicology and molecular docking, offers a valuable computational framework for generating hypotheses regarding perfluorooctanesulfonic acid (PFOS) exposure and its potential contribution to inflammatory bowel disease (IBD) pathogenesis. The identification of core targets such as albumin (ALB), epidermal growth factor receptor (EGFR), FOS, JUN, and interleukin (IL-10), and their proposed involvement in disrupting the mucosal repair-inflammation regulation oxidative stress pathway, provides a crucial starting point for understanding this complex environmental health challenge. However, several points warrant further discussion. We note that this study is compliant with the TITAN Guidelines 2025 – governing declaration and use of AI[2].

First, the *in vitro* findings regarding PFOS and its impact on NCM460 human colon epithelial cells require careful interpretation. The use of a simplified 2D NCM460 monoculture system inherently limits the study of IBD’s complex pathophysiology. It fails to replicate the intricate multicellular environment of the gut mucosa, which includes various epithelial cell types, immune cells, the extracellular matrix, and the vital gut microbiome. This omission limits the exploration of crucial immune–epithelial crosstalk and the full spectrum of chronic inflammatory responses pertinent to IBD. Although computational analysis identified immune pathways such as T-cell receptor signaling and IL-10 as potential targets, the NCM460 cell line, being exclusively epithelial, cannot adequately model these immune-centric mechanisms[3]. Furthermore, the 4-µM PFOS concentration used, while effective in inhibiting cell proliferation, significantly exceeds typical human plasma concentrations (1–30 ng/mL)[4]. This substantial difference poses a translational challenge, making it difficult to extrapolate findings from high-dose *in vitro* or animal models to chronic, low-level human exposure.

Secondly, beyond individual molecular interactions, a deeper systems-level understanding of pathway interplay and feedback loops is imperative. The proposed synergistic disruption of the mucosal repair-inflammation regulation oxidative stress pathway in IBD is likely governed by intricate positive and negative feedback mechanisms. For instance, oxidative stress can trigger NF-κB activation, creating a self-reinforcing loop that perpetuates inflammation[5]. Similarly, if PFOS reduces anti-inflammatory IL-10 production, this could unleash pro-inflammatory cascades. Advanced systems biology modeling, integrating multi-omics data, is essential to simulate these dynamics and identify critical nodes for intervention, moving beyond isolated components to a holistic understanding of system perturbation.

Thirdly, the inherent nature of computational predictions necessitates rigorous experimental validation to bridge the gap between *in silico* insights and confirmed biological mechanisms[6]. While molecular docking can predict binding interactions and the study utilized a Cellular Thermal Shift Assay to demonstrate the interaction between PFOS and ALB, further comprehensive direct binding assays are indispensable to confirm PFOS’s affinity and specificity for all identified core targets.

To conclude, Wang *et al*’s study provides a strong foundation[1]. Addressing the aforementioned points would further deepen our understanding of the mechanisms underlying PFOS-induced IBD.

## Data Availability

All data are included in the article.
